# Comparison of Complicated and Simple Guiding Templates in Mandibular Reconstruction Using Vascularized Iliac Crest Flap

**DOI:** 10.1155/2019/7496538

**Published:** 2019-06-26

**Authors:** Mei Zho, Zhe Shao, Yuxi Zhu, Bing Liu, Tianfu Wu

**Affiliations:** ^1^The State Key Laboratory Breeding Base of Basic Science of Stomatology (Hubei-MOST) & Key Laboratory of Oral Biomedicine Ministry of Education, School & Hospital of Stomatology, Wuhan University, China; ^2^Department of Oral and Maxillofacial Surgery, School and Hospital of Stomatology, Wuhan University, Wuhan 430079, China; ^3^Glasgow Dental Hospital and School, Scotland, UK

## Abstract

**Objective:**

This study aims to compare the degree of accuracy achieved in mandibular reconstruction between complicated guiding templates (CGT) and simple guiding templates (SGT), to evaluate the necessity to spend more time to design complicated templates prior to surgery.

**Methods:**

The preoperative virtual surgery plan (VSP) was used to simulate the osteotomy and accurate mandibular reconstruction strategy. Then the guiding templates were designed and printed to transfer the VSP into the real operation. Between July 2013 and November 2014, we used the SGT in 13 L-type mandibular defect reconstructions utilising vascularized iliac crest bone (VICB). From March 2015 to March 2018, we used CGT in 14 L-type mandibular defects, also reconstructing with VICB. The indicators of mandibular symmetry, midline deviation, alveolar height loss, bone conjunction gap, and operation time were analyzed and compared between the two groups.

**Results:**

The overall bone graft success rate was 100% (27/27) between all patients. The SGT and CGT groups showed similar symmetry (1.01 ± 0.03 vs. 1.03 ± 0.04,* P* = 0.11) and mandibular midline displacement (1.0 ± 0.7 mm vs. 1.2 ± 0.8 mm,* P*=0.29). The CGT group showed less alveolar height deficiency than the SGT group (3.0 ± 2.4 mm vs. 7.8 ± 6.8 mm,* P*=0.01) and lesser bony conjunction gap between the graft and the mandible (1.6 ± 0.7 mm vs. 2.4 ± 1.2 mm,* P* = 0.02). The average operation time was significantly lower in the CGT group than in the SGT group (340.5 ± 74 min vs. 391.9 ± 41.7 min,* P* = 0.02).

**Conclusion:**

In the simple mandibular reconstruction, the time-consuming CGT did not significantly improve the symmetry and midline displacement compared to SGT, but it demonstrated less reduction (increased preservation) in alveolar height and decreased the size of the bone conjunction gap. And in addition, CGT also reduced the average operation time and simplified intraoperative procedures compared with SGT.

## 1. Introduction

Mandibular defects, caused by benign and malignant tumours in the mandible, can have a detrimental effect on oral function, facial appearance, and social activities [1]. Autologous bone flaps such as vascularised fibula, iliac crest bone, and scapula are the best means of reconstructing such defects [2–5]. Since the morphology of these bones is quite different to that of the mandible, recently the VSP and guiding templates have often been used to improve the efficiency and accuracy of reconstruction [6]. With the popularity of VSP, the design of guiding templates tends to be increasingly complex, delicate, and serialized. However, it is unclear whether more complicated guiding templates are conducive to increased accuracy and efficiency in the final operation.

In view of this, we analyzed and compared the application difference between the groups of complicated guiding templates (CGT) and simple guiding templates (SGT). Due to the fact that the variations in the types of defect and of bony grafts required different templates, it was difficult to compare the preparation of the guiding templates. Therefore, in order to compare the accuracy and efficiency of simple and complicated guide plates, we decided to only select one type of mandibular defect and one bone graft for the reconstruction.

## 2. Materials and Methods

### 2.1. Patients

*Between July 2013 and March 2018*, twenty-seven patients underwent mandibulectomy and acquired an L-type mandibular defect (with Jewer's method), which was subsequently reconstructed with VICB at the Stomatological Hospital of Wuhan University. This retrospective study was approved by the Ethics Committee of School and Hospital of Stomatology, Wuhan University. All patients provided informed consent before inclusion, and all patient-related procedures were performed according to the World Medical Association Declaration of Helsinki (version 2008).

SGT was used for 13 patients CGT for 14 patients during reconstructive surgery. The SGT group included 2 males and 11 females, aged between 23 and 52 years old (32.5 years old on average), with diagnoses of ameloblastoma (*n* = 7), ossifying fibroma (*n* = 2), osteofibrous dysplasia (*n* = 1), recurrent odontogenic keratocyst (*n* = 1), odontoma (*n* = 1), and dentinogenic ghost cell tumour (*n* = 1). The CGT group comprised 8 males and 6 females, aged between 14 and 46 years (31.2 years old on average), with diagnoses of ameloblastoma (*n* = 7), recurrent odontogenic keratocyst (*n* = 2), cementum dysplasia (*n* = 1), odontogenic carcinoma (*n* = 1), intravascular bone malformation (*n* = 1), Odontogenic clear cell carcinoma (*n* = 1), and aneurysmal bone cyst (*n* = 1).

#### 2.1.1. Virtual Surgery Plan and Template Design

The DICOM format data of head and bilateral ilium were imported into Mimics 19.0 software (Materialise, Belgium) to simulate the mandibulectomy and defect reconstruction [7]. Then the Stl type data was imported into Geomagic studio 2013 to draw the templates.

In the cutting template, the complicated guiding template adopted an integrated design, with special design considerations such as mental foramen for accurate attachment, locating hole for repositioning, and saw path reserved for saw loss. These were different from the design of the simple template for cross-sectional guidance. In template for graft bone harvest, the complicated template was added with an in-situ shaping template. In template for reconstruction, the simple template used a prebent titanium plate or similar splint for restoration, while the complicated template was a detachable reconstructive template, able to load the titanium plate. In addition, it also integrated the locating hole consistent with the cutting template for rapid repositioning of residual bone segments.

### 2.2. Surgical Procedures

**Figure 1:**
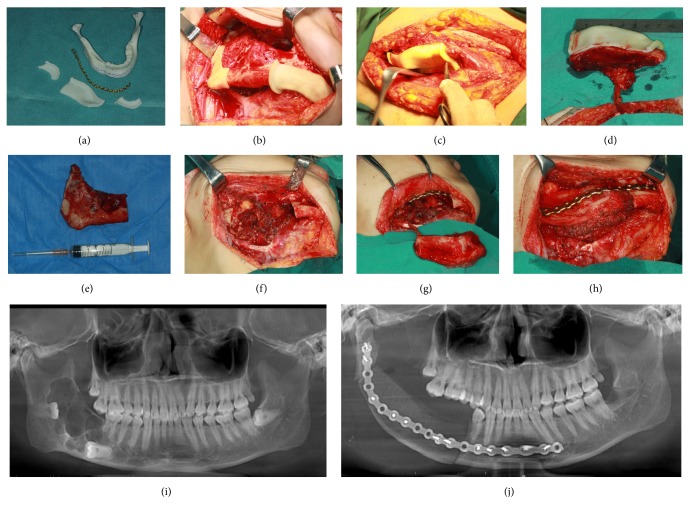
Procedures for the SGT (simple guiding templates). (a) Overview of the cutting guides, prebending titanium plate and reconstructed model. (b) Application of the simple cutting guides onto the surface of mandible, on both side of the lesion. (c) Application of the cutting guide on the iliac area. (d) Harvest of the vascularized iliac bone before pedicle division. (e) The removed mandibular lesion according to the cutting guide. (f) The mandibular defect after mandibulectomy. (g) Restoration of the mandibular continuity by prebending titanium plate and the deep circumflex iliac artery and vein of the bone flap was anastomosed with facial artery and vein. (h) The iliac bone was reshaped and put into the defect and fixed: (i) presurgery panoramic radiograph showed lesion in the right gonion and (j) postsurgery panoramic radiograph showed the lesion was removed and embed by iliac bone.

**Figure 2:**
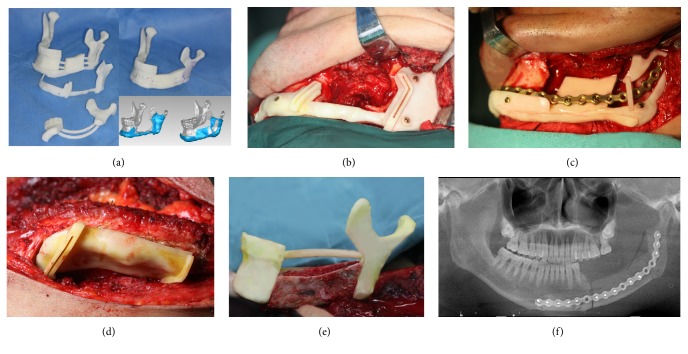
Outlines for the CGT (complicated guiding templates). (a) Overview of the complicated cutting guide, detachable reconstructive template, and in-site reshaping guide. (b) Application of the complicated cutting guide on the surface of mandible. (c) Application of the detachable reconstructive template that integrated with prebending titanium plate to restore the bony parts position after mandibulectomy. (d) Application of the cutting guide in iliac area. (e) Application of the in-site reshaping guide. (f) Postoperative panoramic radiograph showed the defect was reconstructed with iliac bone precisely.

All 27 surgeries were performed in the same group by senior surgeons experienced in VSP-aid surgery. The main steps included the mandibulectomy, repositioning of the residual bone, VICB harvest, remodeling of the the VICB, microvascular anastomosis, and bone fixation. The harvesting of VICB was performed at the same time as the surgery in the mandible. Templates for guidance were used in the corresponding steps (Figures 1 and 2).

### 2.3. Indicators and Statistics

**Figure 3:**
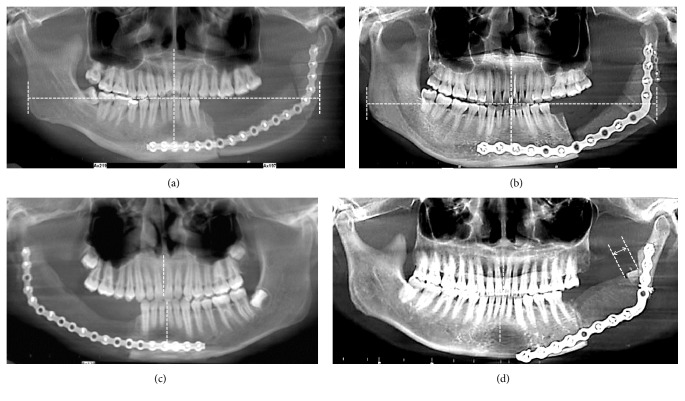
Postsurgery measurement analysis. (a) Measurements of the length of the bilateral mandible. (b) Decrease in the alveolar height and the asymmetry in the left gonial angle. (c) Midline deviation. (d) Midline deviation and gap in the conjunction area that was fitted by a nonvascularised bone.

To compare the reconstructive results between the two groups, we used the following five indicators to evaluate the postoperative outcome. All data was measured thrice and then the average value was used. The panoramic radiographs before and after surgery were adjusted using the anterior tooth to eliminate the size differences. ① The length (*L*) of the unilateral mandible was defined as the horizontal distance between the proximal point of mandibular central incisor and the outermost edge of the mandibular angle (Figure 3(a)). ② The symmetry value (*Sv*) was evaluated using the length ratio between the bilateral mandible (Figure 3(b)). The preoperative* Sv* was pre-*Sv* =* L*_1_/*L*_2_, where* L*_1_ and* L*_2_ were the preoperative ipsilateral and contralateral mandibular lengths, respectively. Similarly, the postoperative* Sv* was recorded as post-*Sv* =* L*_3_/*L*_4_, where* L*_3_ and* L*_4_ were the bilateral lengths of the postoperative mandible, respectively. ③ The midline displacement was the horizontal mandibular midline deviation referred to the maxillary midline before and after operation (Figure 3(c)). Before surgery, the mandibular midline might stay a little to the left- or right-hand side. If the mandibular midline shifted to the same side following surgery, then the degree of mandible displacement was calculated as the preoperative distance minus the postoperative distance. If the midline shifted to the opposite direction, the degree of displacement was calculated as the sum of the two distances. ④ The alveolar height was measured perpendicularly from the superior to inferior aspect of the alveolus. The degree of alveolar height loss was defined as the difference between the design and surgical result (Figure 3(b)). ⑤ The gaps between the graft and the mandible were measured in both anterior and posterior areas, with the maximum value recorded in the statistics (Figure 3(d)). ⑥ The operation time was calculated from the first incision to the final suture of the skin.

## 3. Statistics

All data was measured three times and then the averaged value was used. Each parameter was expressed as mean ± SD. The differences in mandible symmetry, height, displacement, compactness, and operation time between the 2 groups were analyzed using T-test.* P*<0.05 was considered statistically significant.

## 4. Results

### 4.1. CGT Groups Showed Similar Mandibular Symmetry and Midline Displacement with SGT Group

The symmetry change between groups was evaluated by comparing the presurgery (pre-*Sv*) and postsurgery bilateral symmetry values (post-*Sv*). The CGT and SGT groups exhibited significant difference in presurgery symmetry (1.00 ± 0.03 vs. 0.97 ± 0.05,* P* = 0.03), but no significant difference was determined after surgery (1.01 ± 0.03 vs. 1.03 ± 0.04,* P* = 0.11) (Table 2).

The average degree of mandibular midline displacement was 1.0 ± 0.7 mm in the CGT group and 1.2 ± 0.8 mm in the SGT group. The difference between the two groups was not significant (*P* = 0.29). Both results suggested that the complicated template did not improve symmetry achieved in reconstruction of L-type mandible defect.

### 4.2. CGT Showed Less Deficiency of Alveolar Height and Less Bony Gap Than SGT in the Bone Graft

Compared to the presurgery design, on average, the height deficiency in the CGT group was 3.0 ± 2.4 mm, whereas that in the SGT group was 7.8 ± 6.8 mm. The difference between the two groups was statistically significant (*P* = 0.01). The application of CGT in mandibular reconstruction obtained more exact restoration of alveolar height than the SGT.

Horizontally, the average value of the maximum bony conjunction gap was 1.6 ± 0.7 mm (in the range 0.3–2.5 mm) in the CGT group and 2.4 ± 1.2 mm (in the range 1.1–5.4 mm) in the SGT group (*P* = 0.02). The CGT group presented more precise shape of graft.

### 4.3. CGT Group Had Decreased Operation Time Than the SGT Group

We also recorded the total surgery time for all patients. The average operation time was 340.5 ± 74.5 min in CGT group and 391.9 ± 41.7min in SGT group (*P* = 0.02). The main reason for this reduction in time is mostly likely due to the simplification of the steps of bony parts repositioning and titanium plate implantation. Through use of the complicated reconstructive template, these two steps can be completed in one step.

### 4.4. Discussion

It is complicated to reconstruct mandibular defects with vascularized autologous bone, which involves the selection of donor sites, dressing, and restoration of morphology. Compared to the traditional surgeon-dependent operation, today's VSP and personalized guiding template significantly improves the accuracy and efficiency of the procedure [6]. The CT data we used has a layer thickness of 0.625 mm, which could lead to a reduction in accuracy compared to the 0.04 mm used in modern 3D printing. However, this does not affect the application of 3D printing technology in medicine and related research because the difference in precision does not affect the morphological contour, especially the interrelation of anatomical structures, thus allowing it to meet the needs of clinical applications.

Theoretically, by using the VSP and splint-guiding surgery, the graft should precisely match the defect [8]. However, due to the multiple procedures and position transition, deviation is present in the final result [9]. As a result, the design of the guide has a tendency to be more complex, but it is unclear whether more complicated templates facilitate higher quality reconstruction [10]. In this study, through comparison and analysis, it was found that the CGT used in the reconstruction of l-type mandibular defects was only superior in terms of alveolar height, surgery duration, and graft contact but had no significant improvement in the symmetry and midline deviation of the mandible.

We decided to measure the indicators of symmetry, midline deviation, alveolar height, and bony gap, which were more intuitive and easier to measure. We first measured these on the 3D model but found the accurate selection of the reference and linear distance were subjective on the curved surface of the mandible and the graft. Conversely on the panoramic radiograph, which the 3D model projected, these linear measurements were more precise and repeatable. Accordingly, the lengthening or shortening of the unilateral mandible in the sagittal plane or extension in the coronal plane following surgery would be reflected as a length change in the panoramic view.

We compared the degree of postsurgery midline displacement from presurgery position in the two groups and found that CGT did not increase or decrease this deviation. Both groups showed similar-length defects (Table 1) and symmetry. Therefore, together with the statistical data of 27 cases, the results of this analysis were relatively objective and accurate.

However, this result was limited to the L-type mandibular defect, reconstructed with VICB, due to several considerations. Firstly, the variation in mandibular defects (from simple L-, C-, H-type to complex LCL-, HC-, HCL-type), reconstructive method (including ilium, fibula, and scapula), and quantity of bony segments required great differences in the design of guiding templates and thus made it difficult to achieve a valid comparison.

Secondly, due to the great variation in the reconstruction, the time used in the bony remodeling, graft harvest, and wound closing also varied greatly. Therefore, we limited our study to the most common type L-type mandibular defect and reconstruction using one segment of VICB, which only involved four bony interfaces. In contrast, reconstruction of complex mandibular defects, such as secondary LCL-type reconstruction using three segments of VICB, or double folded fibula flap, involve about 8 bone surfaces and require increased adjustment of the bone angle and direction. In this situation, the CGT guide plate is able to show an obvious advantage with regard to time saving, in aspects of bone shaping and contact adjustment.

Our study also showed that for the specific types of defect, the SGT is able to meet requirements in terms of symmetry and midline deviation. While the CGT had no significant improvement in the mandibular symmetry and midline deviation, it increased the efficiency of the operation and reduced the duration of the surgical procedures.

Unlike the previous graft shaping in the recipient area, CGT allowed the graft to undergo fine-grained remodelling in site over time (Figure 2(e)). This improvement reduced the time waste during operation and was also consistent with the result that the operation time in CGT was less than that of the SCT group.

**Figure 4:**
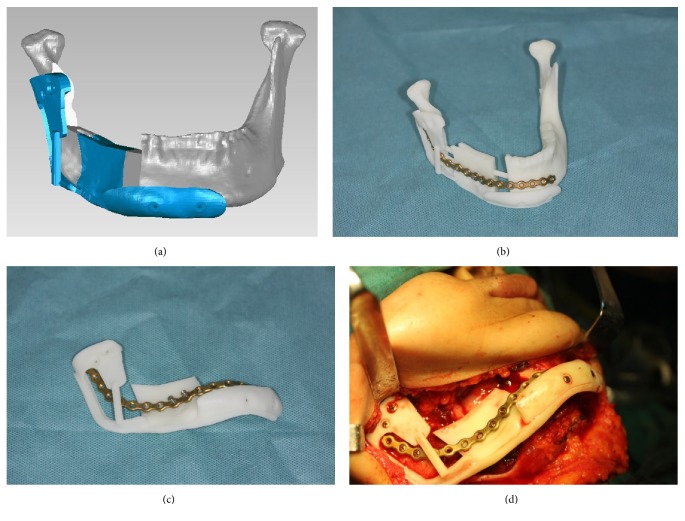
Detachable reconstructed model for the prebending of the titanium plate. The reconstructive template that for bony parts repositioning can be taken down together with the titanium plate so that in the step of using reconstructive template to reposition the bony stumps, titanium plate was simultaneously and naturally fitted on the bone surface.

More importantly, we integrated the prebent titanium plate with the reconstructive template (Figures 4(b) and 4(c)). This method enables accurate determination of the position of the titanium plate alongside the repositioning of the bony parts. This prevents the displacement of the titanium plate, thus improving efficiency and time utilization.

In addition, the above-mentioned CGT design method is a new design which differs from the traditional preplating technique [11]. It is similar to a double plating technique, used in mandibular tumors with bulging of the outer cortex but only uses the outer cortex of the mandible without destruction of the inside muscular attachment. This can also be used in other situations similar to vestibular preplating, lingual preplating, and the Luhr's method, as classified by Marchetti et al. [10]. It can be particularly valuable in situations where the superior portion of the mandibular ramus remains following tumor resection, but there is insufficient space available for the double-plate technique. The expensive titanium plate was not used for prefixation or to exposure of the maxilla for titanium plate fixation by additional incision.

Locating holes were used to record the mandibular parts in the cutting template. During repositioning, the same locating hole information on the reconstructive template was used to restore the position of the bony parts, greatly shortening the time required for reduction of the bone stumps. As this method to restore bony parts was not related to the bony flaps, this method can also be applied into fibula, scapula, and some other potential bony flaps after mandibulectomy. And to this point, we suggest the CGT as a better VSP method which initiatively reduces the surgical deviation before surgery.

## 5. Conclusions

In conclusion, the SGT can achieve similar mandibular symmetry and accuracy compared to CGT. Although the CGT has demonstrated no significant improvement in symmetry and midline deviation, it showed improvement in the aspects of bony integration and iliac bone remodelling and increases efficiency with reduced surgery time. Thus, prior to the widespread use of the personalized 3D-printing titanium, the low price and repeatedly designed functional guiding template is still recommended and can be chosen according to the complexity and priority of the lesion.

## Figures and Tables

**Table 1:** Clinical characteristics of patients.

characteristic	SGT (N = 13)	CGT (N = 14)
male/female	2/11	8/6
Average age (range) (years)	32.5	31.2
Type of Defect	L	L
Length of mandibular defect (mm)	55.6 ± 15	66 ± 16
Benign/Malignant	13/0	13/1

**Table 2:** Comparison of the surgical outcomes in the two groups.

Indicators	SGT (n = 13)	CGT (n = 14)	P value
Post-surgery Symmetry	1.03 ± 0.04	1.01 ± 0.03	0.11
Length Change (mm)	2.6 ± 2.5	2.4 ± 2	0.43
Midline deviation (mm)	1.2 ± 0.8	1.0 ± 0.7	0.29
Alveolar Height Deficiency (mm)	7.8 ± 6.8	3.0 ± 2.4	0.01
Max bony gap (mm)	2.4 ± 1.2	1.6 ± 0.7	0.02
Duration of surgery (min)	391.9 ± 41.7	340.5 ± 74.5	0.02

## Data Availability

The shared measuring data can be found in the online supplementary (available here).

## Supplementary Materials

Supplementary Materials The detailed information of patient's clinical characteristics, measurement values of length, and ratio were included in the supplementary file.
